# Histone proteoform analysis reveals epigenetic changes in adult mouse brown adipose tissue in response to cold stress

**DOI:** 10.1186/s13072-024-00536-8

**Published:** 2024-04-27

**Authors:** Bethany C. Taylor, Loic H. Steinthal, Michelle Dias, Hari Krishna Yalamanchili, Scott A. Ochsner, Gladys E. Zapata, Nitesh R. Mehta, Neil J. McKenna, Nicolas L. Young, Alli M. Nuotio-Antar

**Affiliations:** 1 Verna and Marrs McLean Department of Biochemistry and Molecular Pharmacology, Baylor College of Medicine, Houston, TX USA; 2 USDA/ARS Children’s Nutrition Research Center, Department of Pediatrics, Division of Nutrition, Baylor College of Medicine, Houston, TX USA; 3 Department of Pediatrics, Division of Neurology, Baylor College of Medicine, Houston, TX USA; 4 Department of Molecular and Cellular Biology, Baylor College of Medicine, Houston, TX USA; 5 Center for Precision Environmental Health, Baylor College of Medicine, Houston, TX USA; 6 Jan and Dan Neurological Research Institute, Texas Children’s Hospital, Houston, TX USA

**Keywords:** Brown adipose tissue, DNA methylation, Epigenetics, Gene expression, Histone, Thermogenesis

## Abstract

**Background:**

Regulation of the thermogenic response by brown adipose tissue (BAT) is an important component of energy homeostasis with implications for the treatment of obesity and diabetes. Our preliminary analyses of RNA-Seq data uncovered many nodes representing epigenetic modifiers that are altered in BAT in response to chronic thermogenic activation. Thus, we hypothesized that chronic thermogenic activation broadly alters epigenetic modifications of DNA and histones in BAT.

**Results:**

Motivated to understand how BAT function is regulated epigenetically, we developed a novel method for the first-ever unbiased top-down proteomic quantitation of histone modifications in BAT and validated our results with a multi-omic approach. To test our hypothesis, wildtype male C57BL/6J mice were housed under chronic conditions of thermoneutral temperature (TN, 28°C), mild cold/room temperature (RT, 22°C), or severe cold (SC, 8°C) and BAT was analyzed for DNA methylation and histone modifications. Methylation of promoters and intragenic regions in genomic DNA decrease in response to chronic cold exposure. Integration of DNA methylation and RNA expression datasets suggest a role for epigenetic modification of DNA in regulation of gene expression in response to cold. In response to cold housing, we observe increased bulk acetylation of histones H3.2 and H4, increased histone H3.2 proteoforms with di- and trimethylation of lysine 9 (K9me2 and K9me3), and increased histone H4 proteoforms with acetylation of lysine 16 (K16ac) in BAT.

**Conclusions:**

Our results reveal global epigenetically-regulated transcriptional “on” and “off” signals in murine BAT in response to varying degrees of chronic cold stimuli and establish a novel methodology to quantitatively study histones in BAT, allowing for direct comparisons to decipher mechanistic changes during the thermogenic response. Additionally, we make histone PTM and proteoform quantitation, RNA splicing, RRBS, and transcriptional footprint datasets available as a resource for future research.

**Supplementary Information:**

The online version contains supplementary material available at 10.1186/s13072-024-00536-8.

## Background

Increasing activation of brown adipose tissue (BAT) has been suggested as a therapy to counter obesity and type 2 diabetes [1, 2]. BAT is a mitochondria-rich tissue that is essential for maintaining body temperature through nonshivering thermogenesis (NST). During thermogenic activation, uptake and oxidation of circulating glucose and fatty acid into BAT renders the tissue a biologic “sink” for both nutrients [3]. Altered BAT function in adults is associated with differences in adiposity, glucose homeostasis, insulin sensitivity, and energy expenditure [1, 4–6]. In particular, NST-induced glucose uptake and oxidation by BAT is attenuated in obese and type 2 diabetic patients [7]. While prior studies have established that specific enzymes that modulate DNA methylation and histone modifications are required for the proper differentiation and development of BAT and that only modest chromatin remodeling occurs in BAT in response to reduced thermogenic activity, quantitative data and rigorous comparisons regarding global DNA methylation changes and histone modifications in BAT in response to varying degrees of thermogenic activation in adults is lacking [8–11]. Thus, epigenetic regulation of the thermogenic response in adult BAT remains mostly unexplored and may yield insights into means through which tissue function and consequent beneficial effects on whole-body metabolism may be restored.

Histone modifications are particularly less studied in BAT due to a lack of effective methods for the isolation of nuclei from BAT, acid extraction from fatty tissues, and appropriate methods of analysis. Here, we have developed a method for the first unbiased, top-down mass spectrometry-based proteomic analysis of histone modifications in murine BAT depots. This method allows us to quantitate discrete post-translational modifications (PTMs) and proteoforms of histones H4 and H3 that are associated with different thermogenic responses in BAT from adult mice for the first time. Using this method, reduced representation bisulfite sequencing (RRBS), and findings from RNA-Seq data, we test our hypothesis that the thermogenic response in adult BAT is associated with multiple epigenetic changes that impact gene expression. We integrate our findings for the first-ever comprehensive and unbiased analysis of epigenetic changes associated specifically with BAT thermogenic activation.

## Methods

### Processing RNA sequencing data

Raw sequencing data was downloaded from Gene Expression Omnibus database under accession number GSE96681, using SRA (Sequence Read Archive) toolkit [12]. Sequencing quality and adapter contamination were assessed using FastQC v0.11.9 [13]. Overall quality was determined to be satisfactory, and raw reads were aligned to the genome index using STAR v2.7.9a [14]. The STAR genome index was created using raw FASTA and annotation files downloaded from the GENCODE portal for mouse genome build GRCm38 release V23. Summary of read and alignment quality were generated using MultiQC v1.12 [15].

#### Differentially expressed genes

Gene expression values were computed as the number of reads aligned per gene, using STAR—quantMode GeneCounts. Raw counts were normalized, and genes with an average read count < 50 across all samples were excluded from the differential analysis. The analysis for differential gene expression was carried out using DESeq2 [16]. A false discovery rate (FDR) cut-off of 0.05 and fold change cut-off of 20% (− 0.263 ≤ log2(FC) ≥  + 0.263) were used to identify differentially expressed genes.

#### Differential splicing analysis

Alternative splicing events were quantified and classified using rMATS [17]. Alignment files (BAM) and the GENCODE reference annotation (GTF) for mouse genome build GRCm38 release V23 were used. rMATS classified splicing events into 5 categories: skipped exons, retained introns, mutually exclusive exons, alternative 5′ and 3′ splice sites. An FDR cut-off of 0.05 and an inclusion level difference cut-off of less than − 0.2 or greater than 0.2 were used to screen for statistically significant changes.

### Mammalian phenotype ontology analysis

Genes mapping to MPO phenotypes were retrieved from the Mouse Genome Database [18]. We retrieved a unique set of nodes (n = 15) mapping to the Mammalian Phenotype Ontology terms “impaired adaptive thermogenesis” (MP:0011049) and “abnormal circadian temperature homeostasis” (MP:0011020) and designated these “thermoregulatory nodes” [19]. We then used the hypergeometric test to evaluate the enrichment of thermoregulatory nodes among nodes with the strongest transcriptional footprints among cold challenge-induced gene sets. The universe was set at the total number of unique nodes included in the HCT intersection analysis (n = 691).

### Consensus transcriptional regulatory network analysis

High confidence transcriptional target (HCT) intersection analysis of gene sets has been previously described [20–23]. Briefly, consensomes are gene lists ranked according to measures of the strength of their regulatory relationship with upstream signaling pathway nodes derived across numerous independent publicly archived transcriptomic or ChIP-Seq datasets [24]. To generate mouse ChIP-Seq consensomes, we first retrieved processed gene lists from ChIP-Atlas, in which genes are ranked based on their mean MACS2 peak strength across available archived ChIP-Seq datasets in which a given pathway node is the IP antigen [25]. We then mapped the IP antigen to its pathway node category, class, and family, and organized the ranked lists into percentiles to generate the mouse node ChIP-Seq consensomes [24]. Genes in the 95th percentile of a given node consensome were designated high confidence transcriptional targets (HCTs) for that node and used as the input for the HCT intersection analysis using the Bioconductor GeneOverlap analysis package implemented in R. *P*-values were adjusted for multiple testing by using the method of Benjamini and Hochberg to control the false discovery rate as implemented with the p.adjust function in R, to generate *q*-values [26]. Evidence for a transcriptional regulatory relationship between a node and a gene set was represented by a larger intersection between the gene set and HCTs for a given node than would be expected by chance after FDR correction (*q* < 0.05).

### Mice and dissection

Immediately after weaning at 3 weeks of age, male wildtype C57BL/6J littermates were housed under thermoneutral (TN, 28°C) or mild cold/room temperature (RT, 22°C) conditions, with an additional cohort of RT-housed mice switched to housing under severe cold (SC, 8°C) conditions for two weeks, starting at 8 weeks of age. All mice were housed in groups of 2–3 per cage, with a 12 h light/12 h dark cycle, and were provided adequate bedding and nesting material and unrestricted access to water and a standard chow diet (PicoLab 5V5R) throughout the study. 10-week-old ad lib-fed mice were euthanized between zeitgeber time ZT3–ZT6, and tissues were dissected and weighed after cardiac perfusion with 20 mL phosphate-buffered saline. Tissues were snap-frozen in liquid nitrogen and stored at − 80°C immediately after dissection. All animal studies were approved by the Baylor College of Medicine Institutional Animal Care and Usage Committee.

### Body composition

At the end of the study, total fat and lean masses were measured for ad lib-fed mice using an EchoMRI Whole Body Composition Analyzer (Echo Medical Systems) located in the Mouse Metabolic Research Unit in the US Department of Agriculture/Agricultural Research Service (USDA/ARS) Children’s Nutrition Research Center at Baylor College of Medicine. For analysis of body composition, nonfasting glucose levels, and body and tissue weights, statistical analysis was performed using GraphPad Prism software (v8.4). One-way analysis of variance (ANOVA) with Tukey posthoc testing was conducted, and data are presented as mean ± SEM, with *p* < 0.05 considered statistically significant.

### Blood glucose measurement

Immediately before sacrifice, mice were retroorbitally bled, and nonfasting glucose was measured using a One Touch Ultra 2 glucose meter and test strips (LifeScan).

### Reduced representation bisulfite sequencing

Genomic DNA was extracted from 0.020 to 0.025 g BAT samples obtained from four mice each from TN, RT, and SC groups using a PureLink Genomic DNA Mini Kit (ThermoFisher Scientific) according to manufacturer protocol. Sample libraries were generated from 150 ng genomic DNA and bisulfite conversion was performed using the Ovation RRBS Methyl-Seq System 1–16 with TrueMethyl oxBS kit (Tecan) per manufacturer protocol. After cleanup, library concentration was measured using a Qubit Fluorometer, and fragment distribution was checked using a 2100 Bioanalyzer high sensitivity chip.

Sequencing at a depth of 50–65 million reads per sample was carried out on an Illumina Nextseq 550 System using a NextSeq 500/550 High Output Kit v2.5 (150 Cycles) (Illumina), per manufacturer protocol for 75 bp paired-end sequencing. Libraries were run on an Illumina Nextseq 550 instrument using the MetSeq Primer 1 (NuGEN) mixed with the Read 1 primer (Illumina) according to the Ovation RRBS Methyl-Seq System 1–16 protocol for the first read and the Read 2 primer (Illumina) for the second read, along with standard Illumina indexing primers. Multiplexed samples were spiked with 10% PhiX Control v3 Library calibration control for run quality monitoring. After the run, FASTQ files were generated using Basespace software (Illumina).

#### Data processing

Raw read sequencing quality and adapter contamination were assessed using FastQC v0.11.9 [13]. Overall quality was determined to be satisfactory, and raw reads were aligned to the bisulfite genome using Bismark v0.23.1 [27]. The Bismark bisulfite genome was prepared using the GENCODE raw FASTA file for mouse genome build GRCm38 release 23 and the bowtie2 v2.3.5.1 aligner [28]. Alignments were saved as binary format (BAM) files and used to extract methylation calls for CpG, CHG, and CHH contexts using bismark_methylation_extractor. A comprehensive methylation coverage file was also created, detailing the methylation percentage at each base.

#### Differentially methylated regions

Bismark coverage files were used to generate an object compatible with methylKit v1.20.0 [29], summarizing base-level methylation counts for each sample. Only bases with a minimum read coverage of 10 were retained, and then normalized using a scaling factor derived from differences between median coverage distributions between samples. Methylation counts were summarized over the promoter and gene body regions, and differentially methylated promoters and genes were identified using an adjusted *p*-value (*q*-value) cutoff of 0.05.

#### Functional analysis

Functional enrichment analyses were performed on gene lists of interest using WebGestalt (WEB-based GEne SeT AnaLysis Toolkit) with an FDR cutoff of 0.05 [30]. Minimum number of genes per category was set to 5. Extracted enrichments were then visualized using GoPlot [31].

### Histone isolation

One brown adipose tissue (BAT) and one liver datapoint was achieved for each organism from ~ 0.1 g BAT and ~ 0.2 g liver. BAT samples were homogenized with 1 mL nuclear isolation buffer (NIB) + 0.3% nonyl phenoxypolyethoxylethanol (NP-40) detergent with appropriate epigenetic inhibitors (sodium butyrate, microcystin-LR, 4-benzenesulfonyl fluoride hydrochloride (AEBSF), and dithiothreitol (DTT)) using a Dounce homogenizer. Nuclei isolation was performed as previously described with optimizations of two NIB + NP-40 incubation steps and two NIB-only washes with a centrifuge duration of 10 min at 20,000×*g* [32, 33]. Liver samples were homogenized with 1 mL NIB + 1% NP-40 with appropriate epigenetic inhibitors listed above using a Dounce homogenizer. Nuclei isolation was performed as previously described with three NIB-only washes and the centrifuge duration to 10 min at 20,000×*g* [32]. Acid extraction was performed as previously described [32]. Isolated histones were resuspended in 85 µL 5% acetonitrile, 0.2% trifluoroacetic acid then histone families were separated by offline high-performance liquid chromatography (HPLC) as described in Holt et al*.* [32].

### Histone H3 and H4 mass spectrometry method

#### Histone family offline chromatographic separation

Histone H3 elutes between 42–52 min and histone H4 elutes between 34 and 36.5 min with the offline HPLC method used, as previously described [32, 33]. H3 variants (H3.1, H3.2, H3.3) and H4 fractions were concentrated to dryness with a vacuum centrifuge concentrator (Savant™ SPD131 SpeedVac, Thermo Scientific, Waltham, MA). For histone H3, a standard curve *µg H3* = *(Peak area – 2.6558)/14.221* was used to calculate the mass. Each H3 replicate was resuspended with 10 µL ammonium acetate for digestion with 1:10 mass:mass GluC (Sigma-Aldrich #10791156001) at 37 °C for 1 h. The digestion was quenched by SpeedVac concentration of the sample to dryness.

#### Preparation of samples for MS and online chromatography

For final dilution, H3 was resuspended to 2 µg/µL using MS buffer A (2% acetonitrile, 0.1% formic acid). For histone H4, dried fractions were diluted using µg H4 = (Peak area – 6.0114)/31.215 to calculate the dilution to 200 ng H4/µL MS buffer A (2% acetonitrile, 0.1% formic acid). 1 µL diluted histone H3 or H4 was loaded onto a 10 cm, 100 µm inner diameter C3 column (ZORBAX 300SB-C3 300 Å 5 µm) self-packed into fused silica pulled to form a nanoelectrospray emitter. Online HPLC was performed on a Thermo U3000 RSLCnano Pro-flow system. The 70-min linear gradient using buffer A: 2% acetonitrile, 0.1% formic acid, and B: 98% acetonitrile, 0.1% formic acid is described in Table 1. The column eluant was introduced into a Thermo Scientific Orbitrap Fusion Lumos by nanoelectrospray ionization. Static spray voltages of 2400 V for histone H3 or 1800 V for histone H4 and an ion transfer tube temperature of 320 °C were set for the source.

**Table 1:** Online HPLC parameters for histones H4 and H3

	Histone H4	Histone H3
Sample Volume	1 µL	1 µL
Temperature	4.0 [°C]	4.0 [°C]
Buffer A	2% ACN 0.1% FA	2% ACN 0.1% FA
Buffer B	98% ACN 0.1% FA	98% ACN 0.1% FA
Equilibration	Duration = 5.000 [min]	Duration = 5.000 [min]
Column	10 cm × 100 µm self-packed C3	10 cm × 100 µm self-packed C3
Flow rate	0.200 [µL/min]	0.200 [µL/min]

Linear Gradient	Time (min)	% Buffer B [%]	Time (min)	% Buffer B [%]
	0	29.0	0	4.0
	0.5	29.0	0.5	4.0
	70	35.0	70	15.0
	75	98.0	105	35.0
	80	98.0	110	98.0
	85	5.0	115	98.0
			120	5.0

1 µL of sample was injected per run after resuspension to a standard concentration (200 ng/µL for histone H4; 2 µg/µL for histone H3). The same buffers were used with different gradients (See Linear Gradient section). Histone species were introduced to the Orbitrap Fusion Lumos mass spectrometer using nano-electrospray ionization as they eluted

#### Mass spectrometry analysis of histone proteoforms

The orbitrap MS1 experiment used a 60 k resolution setting in positive mode. An AGC target of 5.0e5 with 200 ms maximum injection time, three microscans, and scan ranges of 585–640 *m*/*z* for histone H3 or 700–1400 *m*/*z* for histone H4 were used. For histone H3, the target precursor selected for MS2 fragmentation included all H3 peaks from GluC peptides and chose the top 6 most abundant *m/z*. ETD fragmentation at 18 ms reaction time, 1.0e6 reagent target with 200 ms injection time was used. MS2 acquisition was performed using the orbitrap with the 30 k resolution setting, an AGC target of 5.0e5, a max injection time of 200 ms, a ‘normal’ scan range, and two microscans. For histone H4, an intensity threshold of 1e5 was set for the selection of ions for fragmentation. The target precursor selected for MS2 fragmentation included all major H4 peaks and chose the top 20 most abundant *m/z*. ETD fragmentation at a 14 ms reaction time, 5.0e5 reagent target with 200 ms injection time was used. MS2 acquisition was performed using the orbitrap with the 60 k resolution setting, an AGC target of 5.0e5, a max injection time of 200 ms, a ‘normal’ scan range, and three microscans.

### Mass spectrometry data analysis

For histone quantitation, 3–5 animals were analyzed in each of three different housing conditions, giving n = 11–15 total for each organ/tissue. Two technical replicates were analyzed per sample and averaged to give the measured value for a minimum of three biological data points per group. Raw files were converted to.mzXML. Data processing was performed by a custom analysis suite as previously described [34, 35]. TDMS version 7.04 and Interp 2v16 were used for analysis. Search parameters include a 3.4 Da window for MS1, 10.0 ppm tolerance for c and z fragment ions. PTMs considered for histone H4 include fixed N-terminal acetylation and variable K5ac, K8ac, K12ac, K16ac, K20me1/2/3, and K31ac. PTMs considered for histone H3.2 include variable K4me1/2/3, K9ac, K9me1/2/3, K14ac, K18ac, K23ac, K27ac, K27me1/2/3, and K36me1/2/3. This data analysis approach has been used extensively in other studies in our lab to reveal quantitative changes in histone proteoforms [33, 34, 36–40]. This approach has also been validated against reverse phase protein array based quantitation [40]. For analyses of BAT versus liver histone modifications, we conducted multiple unpaired t-tests using the false discovery rate approach, and discoveries were determined using the two-stage linear step-up procedure of Benjamini, Krieger and Yekutieli. For all other analyses, Welch’s 2-tailed t-tests or one-way ANOVA with Tukey posthoc testing were used when comparing two or three groups, respectively. *Q-*, *p-*, or *p*_*adj*_*- *values less than 0.05 were considered statistically significant.

#### Fold- and absolute change

A fold change cutoff of |1.5| was also used; however, absolute change was also used to inform decisions on biological significance. Unlike many proteomic methods, we accurately measure absolute change. In our experience, it is not uncommon for large absolute changes to result in small-fold changes yet have profound biological significance. For example, a histone PTM that decorates 50% of the entire genome that increases to 70% results in a change to an astounding 20% of the genome but is less than 1.5-fold. Thus, consideration of both fold change and absolute change is insightful and represented as independent metrics.

### Experimental design and statistical rationale

Unless otherwise stated, experiments were designed with an N of 3–5 per group (BAT or liver from mice housed at TN, RT, and SC). The data obtained was interpreted collectively for a robust understanding of the system. Rigor was enhanced through multiple complimentary methods to understand epigenetic changes during BAT thermogenesis. Various parametric statistics and corrections were used and are described above. Generally, one-way ANOVA with Tukey post-hoc testing or Welch’s two-tailed t-tests were used with *p* < 0.05 considered statistically significant. *P*_*adj*_ or *q* values are calculated where appropriate, namely with consensus transcriptional regulatory network and RRBS analyses and comparisons between tissue histone modifications at each temperature.

## Results

### Analysis of RNA-Seq data reveals housing temperature-dependent effects on genes involved in epigenetic regulation

Acclimation and BAT remodeling in response to ambient temperatures occurs when mice are housed under constant temperatures for at least 7 days [41]. Additionally, prior studies have established that standard laboratory housing of mice at 20–24 °C is a chronic mild cold condition that stimulates NST [41–43]. We reanalyzed and further interrogated publicly-available RNA-Seq data and noted significant housing temperature-induced changes in the expression of genes encoding DNA methyltransferases, *Dnmt1*, *Dnmt3a*, and *Dnmt3b*, acetyl-CoA-generating enzymes *Acly*, *Acss2*, *Crat*, and *Crot*, and histone-modifying enzymes *Smyd3, Kdm2b, Setd7, Kdm3a, Kdm5b, Kdm5c*, and *Kdm8* (Table 2) [43]. Expression of ten-eleven translocation (TET) enzymes, *Tet2* and *Tet3*, which facilitate demethylation of DNA 5-methylcytosine (5mC), did not significantly differ between TN vs SC conditions (data not shown) [44]. In addition, we noted significantly altered expression of genes involved in one-carbon metabolism, suggesting that there may be housing temperature-dependent effects on the methyl donor pool that also contribute to epigenetic modifications in BAT.

### Regulatory network analysis resolves epigenetic writer footprints in cold challenge-regulated gene sets

**Fig. 1:**
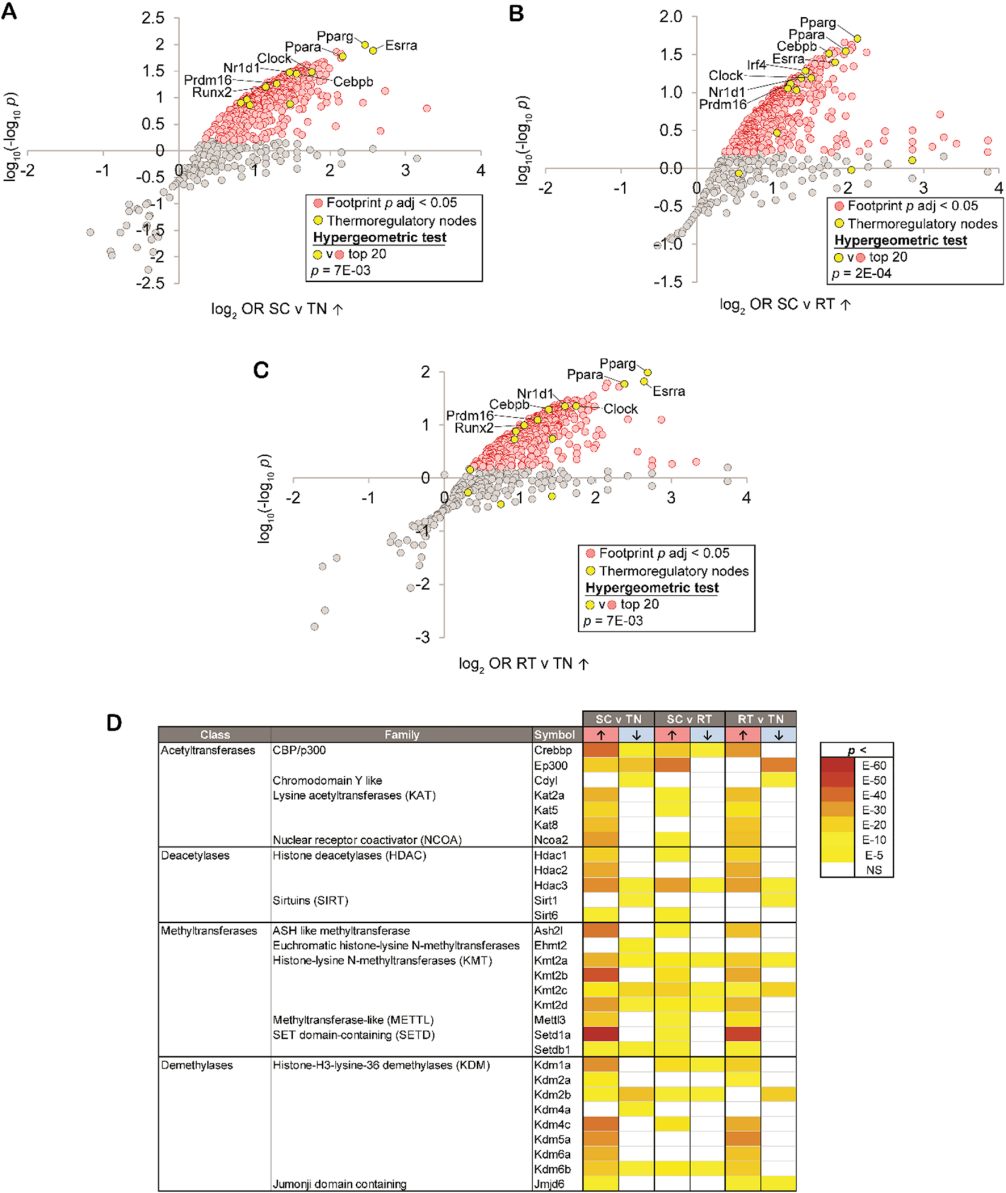
High confidence transcriptional target (HCT) intersection analysis resolves epigenetic writer transcriptional footprints in cold challenge-regulated gene sets. In panels (**A-C**), nodes that have the strongest (higher odds ratio, OR) and most significant (lower p-value) footprints within the indicated cold challenge-induced gene set are distributed towards the upper right of the plot. Scatterplot showing enrichment of nodes with established roles in thermal regulation among nodes that have the most significant intersections with (**A**) SC vs TN-induced genes; (**B**) SC vs RT-induced genes; and (**C**) RT vs TN-induced genes. (**D**) HCT intersection p-values for selected epigenetic writers within cold challenge-induced genes are indicated in the form of a heatmap. HCT intersection analysis was carried out as described in the Methods section. White cells represent p > 0.05 intersections. The intensity of the color scheme is proportional to the confidence of the intersection between HCTs for a particular node and genes induced (red, ↑) or repressed (blue, ↓) in each cold challenge contrast. Lower confidence (higher p) intersections are towards the yellow end of the spectrum and higher confidence (lower p) intersections are towards the brick red end of the spectrum. Full numerical data are in Additional file 1. n = 4 per group for RNA-Seq data. TN: thermoneutral. RT: room temperature. SC: severe cold

High confidence transcriptional target (HCT) intersection analysis uses annotated public ChIP-Seq datasets to predict regulatory roles for transcriptional regulators within gene sets of interest [20–23, 45]. To identify transcription factors and enzymes whose gain or loss of function contributed to the observed cold challenge expression profiles, we subjected genes significantly induced or repressed in each of the three contrasts to HCT intersection analysis (Additional file 1). As validation, we reasoned that our analysis should identify strong footprints within cold exposure-induced genes for nodes encoded by genes whose deletion in the mouse results in deficient thermoregulatory processes. To test this hypothesis, we retrieved a set of nodes (n = 15) mapped to the null phenotype “impaired adaptive thermogenesis” (MP:0011049) or “abnormal circadian temperature homeostasis” (MP:0011020) from the Mouse Genome Database and designated these “thermoregulatory nodes.” We then evaluated the distribution of these thermoregulatory nodes among nodes that had strong regulatory footprints among cold challenge-induced genes in the three contrasts (Fig. 1A–C) [18]. Consistent with the reliability of our analysis, we observe strong enrichment of the thermoregulatory nodes among the top ranked cold-induced footprint nodes in all three experiments. These nodes include the nuclear receptors Pparg, Ppara, and Esrra, in addition to Cebpb and Prdm16, among others [46–50].

We next questioned whether the expression of epigenetic writers in BAT is altered in the response to different housing temperatures. We observe robust (FDR < 0.05) footprints within cold challenge-induced genes for numerous enzymes in the methyltransferase, demethylase, acetyltransferase and deacetylase classes (Fig. 1D). These include nodes with characterized or inferred roles in thermal regulation, such as the acetyltransferases Crebbp/CBP, EP300/p300, and Kat2a/Gcn5; deacetylases such as members of the HDAC and sirtuin families; methyltransferase members of the KMT2 family as well as Mettl3; and the KDM family demethylases [51–60]. We observe particularly strong footprints among cold challenge-induced genes for Setd1a, which has only recently been implicated in thermogenesis [61]. In addition to these nodes, our analysis hints at roles for a number of nodes with no previously resolved role in the response to cold challenge, such as Cdyl, Kat5/TIP60, Kat8, Ash2l, Ehmt2 and Setdb1.

### Establishing a mouse model to interrogate the BAT epigenetic cold response

To investigate epigenetic changes in BAT due to chronic thermogenic activation in response to mild or severe cold housing temperatures, tissues were collected from 10-week-old mice housed under thermoneutral (TN, 28°C), room temperature (RT, 22°C), or, for the last two weeks of the study, severe cold (SC, 8°C) conditions. Our data support prior reports that lower housing temperatures are associated with significant reductions in adiposity, as we observe decreased end body weight, total fat mass, and weight of gonadal white adipose tissue (gWAT) in SC- vs RT- or TN-housed mice [62]. There was no significant effect on nonfasting blood glucose levels, lean mass, or weights of BAT, liver, or spleen in our study (Additional file 7: Table S1). Consistent with previous studies conducted with hamsters and rats, kidney weight was inversely associated with housing temperature [63, 64].

### DNA methylation and gene expression in BAT are altered in response to chronic thermogenic activation

**Fig. 2:**
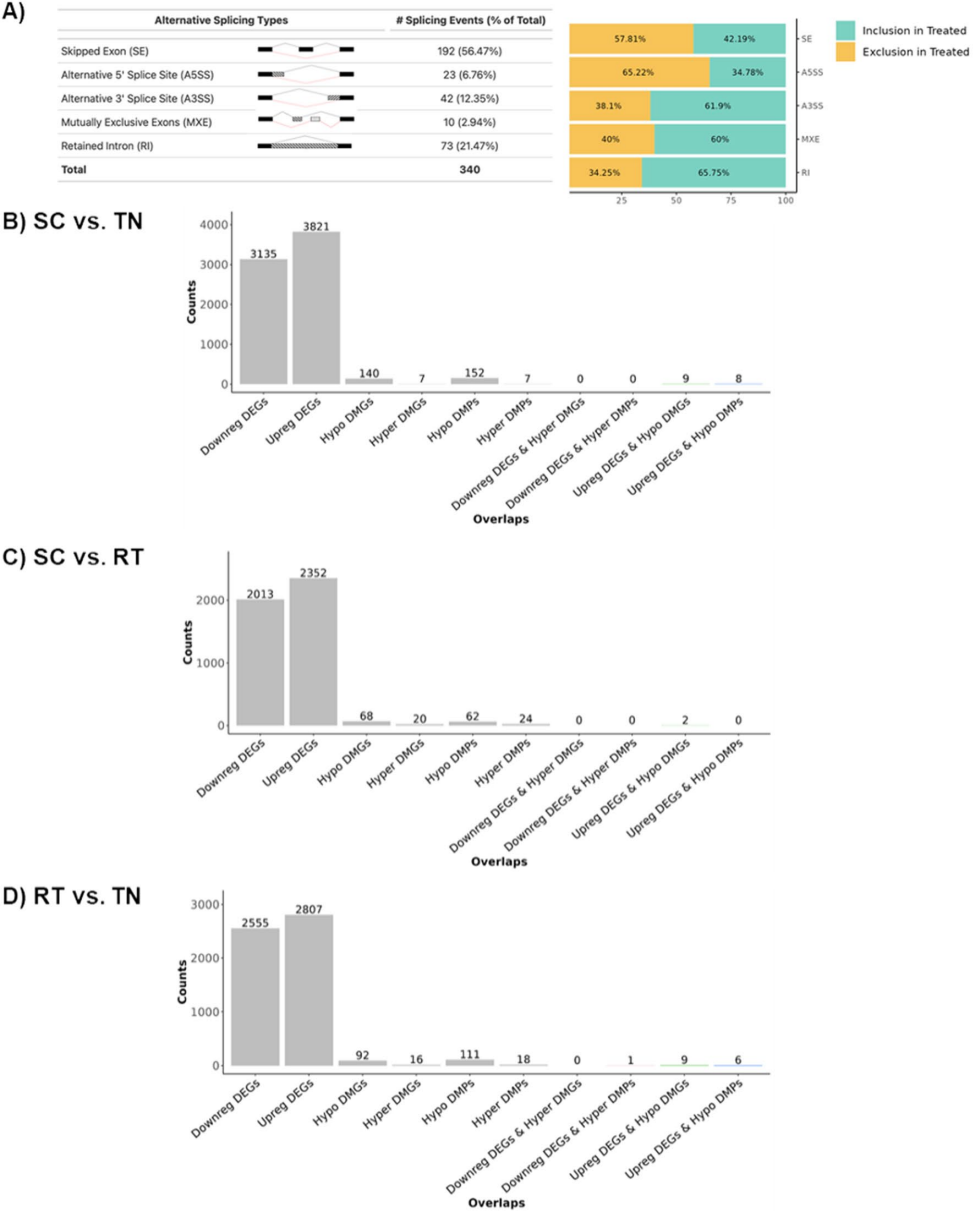
Alternative splice variants and reduced representation bisulfite sequencing (RRBS) data reveal changes in brown adipose tissue associated with housing temperature. (**A**) A comparison of SC vs TN housing RNA-Seq data reveals differential expression of alternate splice variants in BAT. Integrated RNA-Seq and RRBS data for (**B**) SC vs TN housed mice. (**C**) SC vs RT housed mice; and (**D**) RT vs TN housed mice. n = 4 per group for RNA-Seq and RRBS datasets. RRBS data shown are for percent methylation difference >|5|, p_adj_ < 0.05. DEG: differentially expressed genes. DMP: differentially methylated promoters. DMG: differentially methylated genes. TN: thermoneutral. RT: room temperature. SC: severe cold

DNA methylation at the promoters and first intron of genes is inversely correlated with gene expression, whereas intragenic DNA methylation may reduce spurious transcription and regulate the expression of alternate splice variants [65–68]. To date, it is not known whether adaptation to cold induces large-scale epigenetic changes to DNA that impact global expression of genes and their splice variants in BAT. Reanalysis of the RNA-Seq data yielded numerous differentially expressed alternative splicing factors and alternative splice variants for BAT taken from SC- vs TN-housed mice (Table 2, Fig. 2A, and Additional files 2 and 3). To assess whether epigenetic modifications of DNA were occurring in BAT in response to chronic thermogenic activation, we first conducted reduced representation bisulfite sequencing (RRBS) using genomic DNA extracted from BAT samples taken from mice housed under TN, RT, and SC temperature conditions. We noted 111 differentially hypomethylated and 18 differentially hypermethylated promoters; and 92 differentially hypomethylated and 16 differentially hypermethylated intragenic regions in BAT from RT-vs TN-housed mice (Fig. 2B–D). Housing at SC further reduced promoter and intragenic region methylation when compared with TN. There were fewer differentially methylated promoters and intragenic regions in BAT taken from mice housed under SC vs RT conditions. These data show that changes in DNA methylation in BAT are associated with chronic activation of the NST response and that the more severe the chronic cold challenge, the greater the changes to DNA methylation.

**Table 2:** Expression of acetyl-CoA generating enzymes, one-carbon metabolic genes, epigenetic modifiers, and alternative splicing factors is altered in response to chronic thermogenic activation as measured by RNA-Seq

Gene	SC vs TN		SC vs RT		RT vs TN		Category
	Log_2_FC	P_adj_	Log_2_FC	P_adj_	Log_2_FC	P_adj_	
*Acly*	3.69	5.10E−11	1.03	1.19E−02	2.62	2.92E−05	Acetyl-CoA generation
*Acss2*	2.52	2.64E−33	− 0.08	0.77	2.56	1.36E−28	Acetyl-CoA generation
*Crat*	1.14	1.15E−35	0.27	9.10E−05	0.82	6.26E−16	Acetyl-CoA generation
*Crot*	0.43	6.96E−04	0.09	0.44	0.30	1.82E−02	Acetyl-CoA generation
*Slc25a32*	1.47	3.61E−13	0.72	7.85E−03	0.71	1.54E−02	One-carbon metabolism
*Sfxn1*	1.14	5.20E−18	− 0.07	0.59	1.17	1.37E−19	One-carbon metabolism
*Mthfd2l*	0.95	4.60E−15	− 0.05	0.72	0.96	1.00E−15	One-carbon metabolism
*Aldh1l1*	0.91	1.88E−10	− 0.12	0.59	0.99	2.07E−07	One-carbon metabolism
*Tcn2*	0.72	1.82E−16	0.06	0.63	0.61	1.32E−09	One-carbon metabolism
*Mthfd2*	0.68	1.64E−03	1.31	5.77E−12	− 0.67	1.24E−02	One-carbon metabolism
*Sardh*	0.64	5.69E−10	0.00	0.99	0.60	5.28E−04	One-carbon metabolism
*Chdh*	0.60	4.24E−06	0.12	0.31	0.44	1.76E−03	One-carbon metabolism
*Pemt*	0.54	2.33E−03	− 0.32	0.13	0.82	4.06E−05	One-carbon metabolism
*Slc19a1*	0.52	7.30E−04	− 0.24	0.26	0.72	1.50E−04	One-carbon metabolism
*Ahcyl1*	0.46	2.56E−03	0.12	0.24	0.30	0.07	One-carbon metabolism
*Fpgs*	− 0.30	0.07	0.24	0.22	− 0.58	2.12E−03	One-carbon metabolism
*Shmt2*	− 0.31	4.47E−03	− 0.13	0.23	− 0.22	0.08	One-carbon metabolism
*Sfxn3*	− 0.52	2.24E−02	0.40	3.60E−02	− 0.96	3.48E−05	One-carbon metabolism
*Mat2a*	− 0.68	5.56E−08	− 0.08	0.75	− 0.64	1.58E−03	One-carbon metabolism
*Mtr*	− 0.86	7.19E−24	− 0.30	8.94E−03	− 0.61	2.98E−09	One-carbon metabolism
*Shmt1*	− 1.01	1.80E−14	− 0.71	3.35E−09	− 0.34	2.32E−02	One-carbon metabolism
*Aldh1l2*	− 1.36	9.27E−06	−	−	− 1.18	1.31E−04	One-carbon metabolism
*Dnmt1*	− 0.40	7.02E−03	0.28	8.49E−02	− 0.73	1.33E−06	DNA methyltransferase
*Dnmt3a*	− 0.38	1.53E−03	0.04	0.77	− 0.47	1.16E−04	DNA methyltransferase
*Dnmt3b*	− 1.30	2.18E−06	−	−	− 0.75	6.45E−03	DNA methyltransferase
*Smyd4*	1.32	3.28E−16	1.06	6.11E−13	0.21	0.32	Histone modification
*Fcor*	1.23	3.40E−25	− 0.79	2.18E−15	1.98	3.80E−98	Histone modification
*Sirt5*	0.94	4.31E−09	0.00	0.98	0.91	1.59E−06	Histone modification
*Kdm5c*	0.65	1.12E−12	0.24	5.52E−03	0.36	1.02E−04	Histone modification
*N6amt1*	0.62	5.38E−07	0.05	0.75	0.52	8.27E−05	Histone modification
*H1f0*	0.51	1.15E−04	0.16	0.27	0.31	1.68E−02	Histone modification
*Setd7*	0.50	2.28E−03	0.50	3.85E−04	− 0.03	0.90	Histone modification
*Kdm3a*	0.50	8.89E−03	0.55	1.69E−02	− 0.09	0.69	Histone modification
*Kdm8*	0.50	1.06E−02	0.20	0.54	0.27	0.42	Histone modification
*Hdac2*	0.46	4.84E−06	0.12	0.35	0.30	2.12E−02	Histone modification
*Kat8*	0.45	1.23E−04	0.06	0.74	0.36	8.18E−03	Histone modification
*Setd3*	0.41	1.23E−06	− 0.05	0.68	0.42	2.77E−05	Histone modification
*Kat2b*	0.37	4.19E−03	0.15	0.28	0.18	0.20	Histone modification
*Prmt7*	0.24	2.90E−02	0.16	0.16	0.03	0.84	Histone modification
*Kdm2b*	0.13	0.43	0.54	5.61E−03	− 0.45	3.21E−02	Histone modification
*Hdac5*	− 0.07	0.50	− 0.30	2.30E−04	0.19	2.87E−02	Histone modification
*Hdac3*	− 0.16	0.08	− 0.47	3.87E−10	0.27	1.68E−03	Histone modification
*Hdac10*	− 0.24	4.81E−02	− 0.28	3.87E−02	0.00	1.00	Histone modification
*Kdm5b*	− 0.33	1.26E−02	− 0.31	1.74E−02	− 0.06	0.68	Histone modification
*Hat1*	− 0.38	1.32E−03	− 0.26	3.02E−02	− 0.16	0.22	Histone modification
*Chaf1a*	− 0.53	3.24E−02	−	−	− 1.07	1.34E−04	Histone modification
*Smyd3*	− 0.56	3.30E−03	− 0.48	1.85E−02	− 0.12	0.52	Histone modification
*Hdac7*	− 0.64	9.63E−11	− 0.09	0.54	− 0.60	1.62E−07	Histone modification
*Suv39h1*	− 1.08	3.18E−08	− 0.35	0.16	− 0.77	3.52E−04	Histone modification
*Hdac9*	− 1.20	1.16E−06	−	−	− 0.94	2.93E−05	Histone modification
*Igf2bp2*	0.79	3.92E−03	0.93	3.03E−07	− 0.20	0.60	Alternative splicing
*Elavl1*	0.44	1.02E−04	0.23	1.37E−02	0.17	0.15	Alternative splicing
*Ybx3*	0.11	0.37	− 0.19	0.10	0.25	3.62E−02	Alternative splicing
*Ybx1*	− 0.08	0.34	− 0.39	3.03E−05	0.27	6.71E−03	Alternative splicing
*Ctcf*	− 0.13	0.12	0.01	0.90	− 0.19	4.19E−02	Alternative splicing
*Srsf1*	− 0.33	6.75E−04	− 0.32	3.91E−03	− 0.05	0.68	Alternative splicing
*Srsf2*	− 0.33	4.43E−04	− 0.20	0.15	− 0.18	0.18	Alternative splicing
*Srsf11*	− 0.40	5.36E−04	− 0.52	9.59E−10	0.08	0.49	Alternative splicing
*Rbm4*	− 0.45	0.14	− 0.58	3.30E−02	0.10	0.76	Alternative splicing
*Srsf5*	− 0.59	1.70E−06	− 0.48	3.81E−04	− 0.16	0.10	Alternative splicing
*Srsf3*	− 0.62	2.17E−07	− 0.27	6.08E−02	− 0.39	4.10E−03	Alternative splicing
*Srsf7*	− 0.66	4.31E−11	− 0.09	0.50	− 0.61	3.03E−06	Alternative splicing
*Nova2*	− 0.78	2.15E−05	0.04	0.87	− 0.86	1.46E−06	Alternative splicing
*Pspc1*	− 0.80	8.13E−04	− 0.52	3.80E−02	− 0.32	0.30	Alternative splicing

n = 4 per group. Log_2_FC: log_2_ fold change. TN: thermoneutral. RT: room temperature. SC: severe cold

We next sought to identify genes with changes in promoter methylation that are inversely associated with gene expression, suggesting gene expression regulation resulting from altered DNA methylation. We integrated our RRBS and RNA-Seq data from C57BL/6J wildtype mice housed under similar conditions (Table 3; Additional file 3) [43]. In mice housed at RT vs TN, *Car8*, *Ndufb11*, *Vma21*, *Vldlr*, *Lyplal1*, and *Car13 showed* the greatest percent reduction in promotor methylation that was also associated with increased gene expression in BAT (percent methylation difference >|5|; 1.5–1.8-fold expression; *p*_*adj*_ < 0.05). Conversely, increased promoter methylation for *Tpcn2* was associated with decreased expression (0.54-fold, percent methylation difference >|5|; 0.54-fold expression; *p*_*adj*_ < 0.05). SC housing resulted in hypomethylation of additional promoters, including the promoters for *Dlg3*, *Uba1*, *Dnajc14*, and *Tspan31*, for which gene expression was also significantly increased (1.15–2.65-fold, percent methylation difference >|5|; 1.15–2.65-fold expression; *p*_*adj*_ < 0.05). Although we observed significant differential methylation of promoters in BAT from SC- vs RT-housed mice, none were inversely associated with gene expression changes. Taken together, our findings from integrated RNA-Seq and RRBS datasets suggest that differential methylation of DNA at promoters may play a role in the differential expression of genes in response to chronic severe cold in BAT.

**Table 3:** Integration of RRBS data with RNA-Seq reveals genes that may be regulated by altered DNA methylation in response to different housing temperatures

Gene name	SC vs TN				RT vs TN			
	DMPs		DEGs		DMPs		DEGs	
	Meth Diff	P_adj_	Fold Change	P_adj_	Meth Diff	P_adj_	Fold Change	P_adj_
*Tspan31*	− 36.93	1.53E−47	1.20	1.06E−02				
*Dnajc14*	− 34.23	1.67E−28	1.28	1.83E−06				
*Car13*	− 31.99	8.74E−42	2.25	7.28E−19	− 30.66	4.79E−38	1.53	1.24E−07
*Gm33699*	− 25.58	4.08E−34	6.57	2.73E−13				
*Lyplal1*	− 16.43	9.92E−10	2.65	2.59E−28	− 19.18	4.58E−17	1.78	1.43E−06
*Uba1*	− 15.99	4.05E−23	1.25	7.21E−04				
*Dlg3*	− 11.02	1.55E−29	1.29	8.61E−03				
*Vma21*	− 6.75	4.50E−14	1.80	1.48E−14	− 6.03	1.68E−11	1.53	2.66E−08
*Vldlr*	− 6.28	2.40E−08	1.38	5.22E−03	− 6.86	9.66E−14	1.78	1.06E−06
*Ndufb11*	− 5.19	1.47E−17	1.15	3.60E−02	− 5.39	3.89E−23	1.62	3.75E−08
*Car8*					− 5.28	3.30E−03	1.60	7.67E−04
*Tpcn2*					14.30	8.34E−05	0.54	2.90E−03

n = 4 per group for each dataset. Meth Diff: differential methylation level. DMPs: differentially methylated promoters. DEGs: differentially expressed genes. TN: thermoneutral. RT: room temperature. SC: severe cold

### Quantitative, proteoform-level data can be obtained from brown adipose tissue

**Fig. 3:**
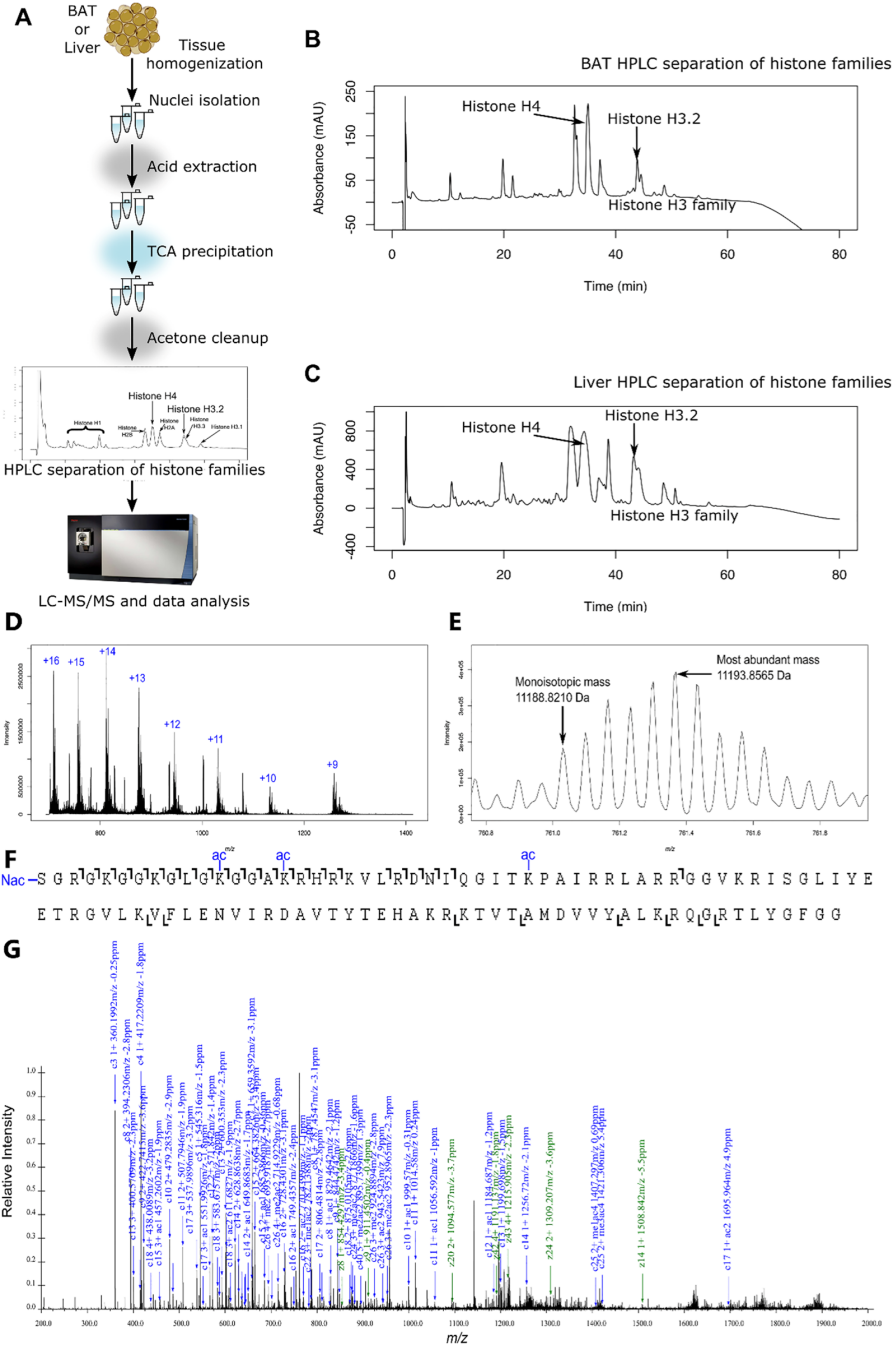
Workflow to obtain histone proteoform data from brown adipose tissue and liver. (**A**) The general workflow includes tissue homogenization, nuclei isolation, acid extraction, HPLC separation of histone families, and LC–MS/MS data acquisition. HPLC separation of histones shows a clean separation of histone families and H3 variants for (**B**) BAT and (**C**) liver. (**D**) MS1 average spectra of histone H4 extracted from BAT from 40 to 80 min show clear + 16 to + 9 charge states matching the m/z of histone H4. (**E**) The MS1 spectrum of 11,193.8565 Da species (761.2961 m/z, charge + 15) matches a mass of H4 with N-terminal acetylation + 0 methylations + 3 acetylations + 0 phosphorylations with less than 10 ppm error. (**F**) The ion map from MS2 fragmentation of species from (**E**) shows unambiguous localization information for the proteoform H4 < N-acK12acK16acK31ac > from a mass change at less than 10 ppm error. (**G**) Annotated MS2 spectrum shows most abundant peaks are from the same proteoform, < N-acK12acK16acK31ac > , with less than 10 ppm error

The capacity to rigorously and sensitively quantitate a wide dynamic range of histone proteoforms has only recently been established [34, 38]. Furthermore, top-down mass spectrometry-based analysis of histone proteoforms has not been previously achieved for BAT in any study to date. A “proteoform” is a protein defined with chemical precision, including how combinations of PTMs co-occur in cis, on single molecules [69]. Thus, quantitation of histone proteoforms accesses the true physiological state and simultaneously provides quantitation of associated discrete attributes such as histone PTMs. To analyze changes in histone modifications in BAT due to different housing temperatures, we first developed histone extraction methods for BAT, building on our protocol for histone extraction from cells [32]. Considering the high lipid content of BAT, we performed a matrix of experiments changing the percent detergent (NP-40, 0.3–1%), the number of times incubated with detergent, the incubation time in detergent, the number of subsequent washes of nuclei, and the duration of centrifugation. Successful changes that were incorporated in this protocol include an additional incubation with detergent, an additional wash of nuclei, and a longer duration of centrifugation compared to our previously published protocol [32]. A summary of histone yield with chosen experiments is shown in Additional file 8: Table S2. Acid extraction was successful with no changes to the protocol. Once histones were isolated, offline HPLC was used to separate histone families and H3 variants, and our published mass spectrometry methods could be used [32, 34]. Our final workflow is shown in Fig. 3A. We obtained adequate offline HPLC separation of histone family members yielding sufficient quantity for LC–MS/MS analysis (Fig. 3B, Additional file 9: Table S3A). This allows for very efficient histone extraction, yielding more histone per gram brown adipose tissue than obtained with livers (Fig. 3C and Additional file 9: Table S3B). Once these histones were prepared and analyzed by mass spectrometry, we obtained high-quality chromatography, localization of PTMs, and quantitation of proteoforms (Fig. 3D–G). Overall, our methodological advancements enable histone isolation from BAT for mass spectrometry analysis. Combined with existing methods, we obtain unbiased histone proteoform quantitation from BAT for the first time.

### Quantitative analysis of histone post-translational modifications and proteoforms

Proteoforms are chemically defined single protein molecules, complete with all sources of variation [69]. This includes how PTMs co-occur, in cis, on the same molecule and on which sequence variant [70, 71]. Thus, quantitative proteoform-level data presents a more complete description of the true physiological state of histone proteins [72]. To distinguish PTMs and proteoforms we use curly brackets “{}” to indicate that the enclosed PTMs are present, and the abundance of all proteoforms that contain those modifications are summed. Angle brackets “ <  > ” indicate that the enclosed PTMs are the only modifications on the histone protein. For example, H3 < K9me2K14acK23acK27me3 > is one of the most common states of histone H3 [73].

### Cold adaptation results in tissue-specific changes in total histone H3.2 and H4 acetylation in BAT, but not methylation

**Fig. 4:**
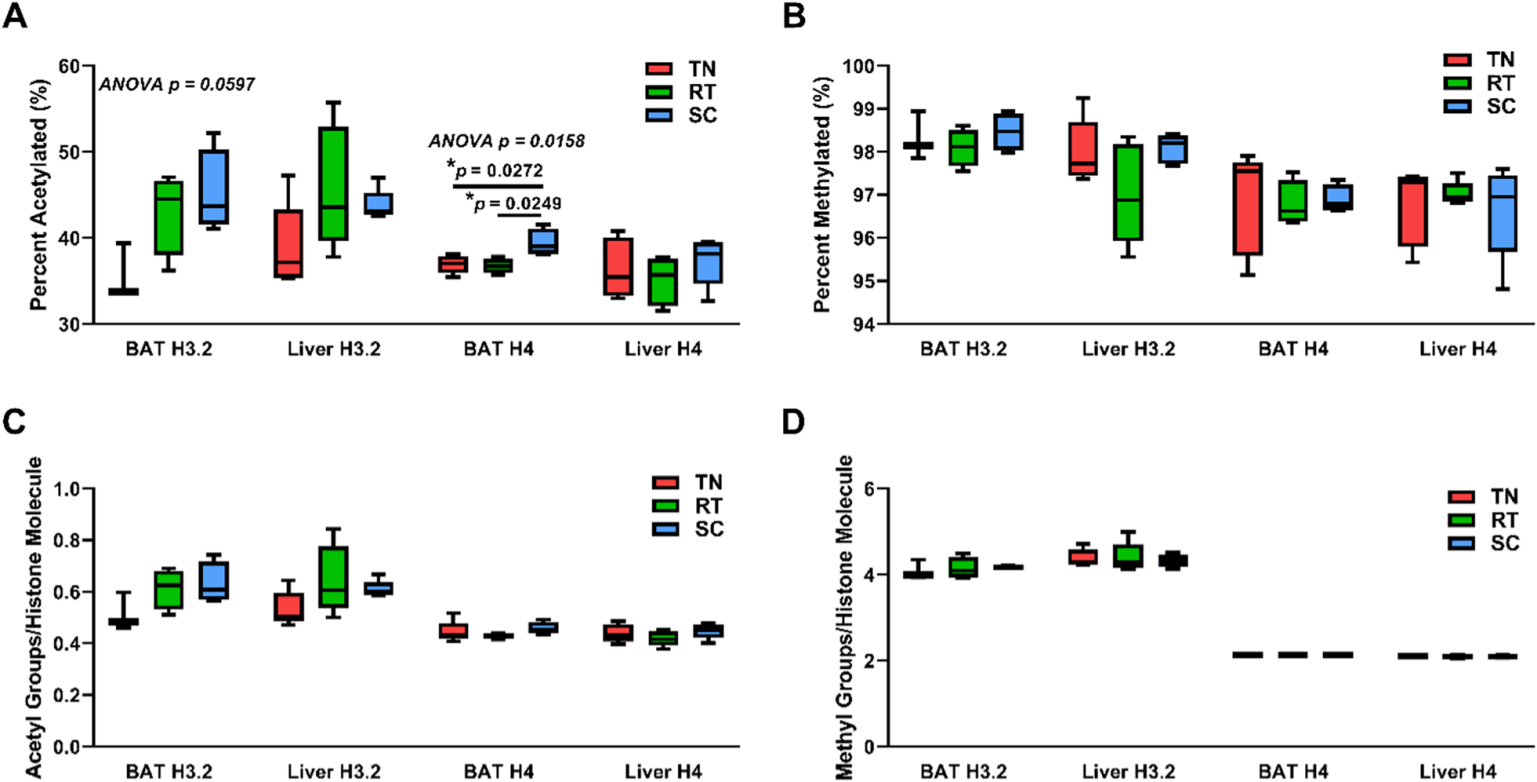
Housing temperature alters bulk histone acetylation, but not methylation, in BAT and has no effect on bulk histone acetylation or methylation in liver. Percent (**A**) acetylated (1 + acetylation) and (**B**) methylated (1 + methylation) histones H3.2 and H4 isolated from liver and BAT. Number of (**C**) acetyl groups and (**D**) methyl groups (K4me2 = 2 methyl groups) per histones H3.2 and H4 isolated from liver and BAT. n = 3–5 per group. One-way ANOVA testing was conducted for all histone data from each tissue comparing TN, RT, and SC housing conditions, and p-values are indicated in each plot. TN: thermoneutral. RT: room temperature. SC: severe cold

The observed housing temperature-dependent differences in the expression of genes involved in acetyl-CoA generation and one-carbon metabolism in BAT suggests that the pool of available acetyl-CoA and methyl donor (*S*-adenosyl methionine, SAM) for histone modification may also be influenced by the degree of thermogenic activation (Table 2). Therefore, we sought to determine the effect of housing temperature on bulk histone acetylation and methylation in BAT. To understand the unique basal epigenetic state of BAT, we also analyzed histones from liver samples taken from the same animals for comparison. First, we compare histones that are methylated or acetylated to any degree in BAT and in liver reference tissue. Generally, these levels are similar between BAT and liver and vary insignificantly between housing temperature with the exception of BAT histone H4 acetylation. Roughly 35–40% of histone H4 is acetylated to any degree and 35–55% of histone H3.2 is acetylated to any degree, Fig. 4A. Acetylations are important modifications for histone H4; however, the most abundant H4 proteoforms are devoid of acetylation. The most abundant H4 proteoforms are < K20me2 > and < K20me3 > and are found on approximately 40 and 20% of histone H4. The percent of histone with one or more acetylations changes for both histone H3.2 and H4 in BAT (Fig. 4A). Histone H3.2 with at least one acetylation is more abundant in BAT at SC compared to TN (45.2 and 35.6% H3.2 that has at least one non-N terminal acetyl group).

Histone H3.2 acetylation in BAT is more abundant at RT compared to TN (43.1 and 35.6% acetylated). Histone H4 acetylation is also the most abundant in BAT at SC compared to TN (39.4 and 36.9% acetylated, *p* = 0.03) and SC compared to RT (39.4 and 36.8% that is acetylated, *p* = 0.02). However, there is no significant effect of housing temperature on bulk histone acetylation in liver. There are also no significant differences between tissues with histone H3.2 or H4 acetylation at each housing temperature. Roughly 95–98% of histone H4 is methylated to any degree and 96–99% of histone H3.2 is methylated to any degree, Fig. 4B. Histone H3.2 from BAT is uniquely almost always methylated to any degree and ranges from 97-99% of total H3.2. The percent of histones with at least one methylation did not change significantly with housing temperature or tissue type (Fig. 4B). As noted above, these high stoichiometric occupancies are expected because H3 is physiologically hyper-modified and we are considering multiple sites of methylation in bulk. Our analysis approach allows for absolute quantitation and more directly reflects the stoichiometric use of the acetyl-CoA pool, the number of acetyl or methyl groups per histone molecule, and is shown in Fig. 4C and D, respectively. This was calculated for each biological replicate by multiplying the abundance of each proteoform by the number of acetylations or methylations on the histone molecule. These values were added and then divided by 100 (because our data are represented as percent of total histone) to reflect the average number of acetylations or methylations per histone molecule. Importantly, histone H4 is mostly unacetylated due to the dominance of < K20me2 > and < K20me3 > abundance (Fig. 4A and Additional file 4). Histone H3.2 acetylation follows a similar trend, in which BAT at SC shows the highest acetylation levels, 0.63 acetylations per histone H3.2 molecule, except for liver at RT, with 0.65 acetylations per H3.2. There are large differences in acetylations per histone H3.2 molecule, with a 0.12 difference between SC and TN housing and a 0.10 difference between RT and TN housing. Histone H4 acetylation is highest in BAT from mice housed at SC, with 0.46 acetylations per histone H4 molecule, and second highest in liver from mice housed at SC, with 0.45 acetylations per H4 molecule. Taken together, our data show for the first time that bulk changes to histone acetylation occur in BAT with cold adaptation and are tissue-specific.

### Brown adipose tissue exhibits different histone PTMs and proteoforms than liver

**Fig. 5:**
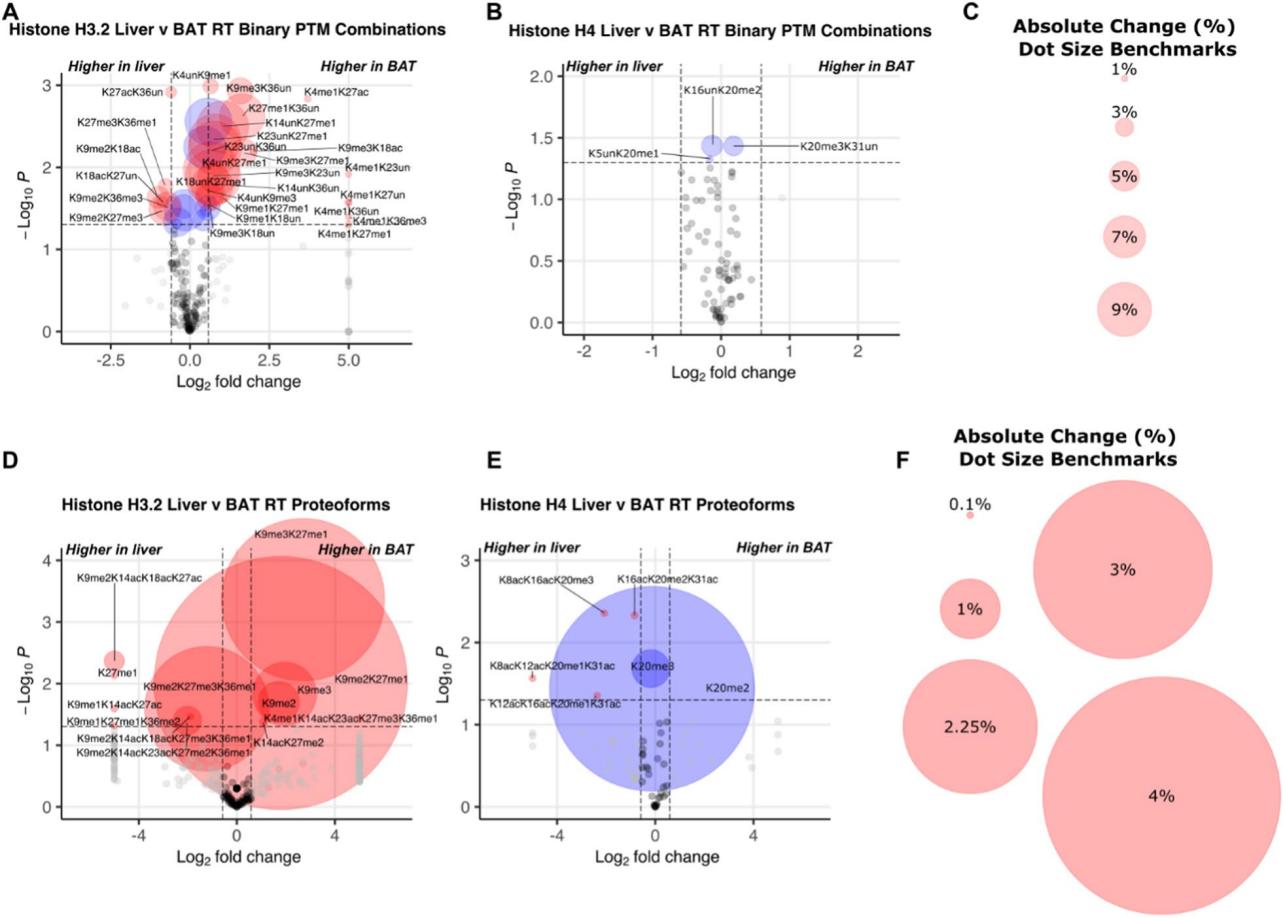
Under normal room temperature housing conditions, BAT histone H3.2 and H4 post-translational modifications and proteoforms are distinct from those observed in liver. (**A**) Histone H3.2 and (**B**) histone H4 binary PTM combinations in BAT and liver. (**C**) Dot size references for binary volcano plots (**A**, **B**). (**D**) Histone H3.2 and (**E**) histone H4 proteoforms from BAT and liver. (**F**) Dot size references for proteoform volcano plots (**D**,**E**). P-values were calculated using Welch’s t-test. Volcano plots show cutoffs of |1.5| for fold change and p < 0.05. Red dots represent both p < 0.05 and fold change >|1.5|; blue dots represent p < 0.05 and fold change <|1.5|; grey dots represent p > 0.05 and fold change >|1.5|; black dots represent p > 0.05 and fold change <|1.5|. n = 3–5 per group. RT: room temperature

We next sought to determine whether histone PTMs and proteoforms are different in BAT versus liver. Here, we focus on significant differences at RT between tissues for discrete PTMs, binary PTM combinations, and proteoforms (Fig. 5 and Additional file 4). However, the highest number of significant differences between tissues is at SC. In general, K9me3 and K36un and their combinations are more abundant in BAT compared to liver. Exceptions include {K27ac,K36un} at RT and {K27me3,K36un} at SC. At RT, BAT histone H3.2 shows significantly higher levels of PTMs K9me1, K9me3, K27me1, and K36un. Many differences are also observed between tissues for binary modifications and proteoforms in histone H3.2 (Fig. 5A, D). Because of our absolute quantitation, we can calculate the difference—not just fold change—in the abundance of PTMs or proteoforms. This difference is indicative of the percent of the genome that is affected by the change in this proteoform (percentage point, pp). For example, H3.2 < K9me2K27me1 > is 1.7% abundant in the liver and 6.0% abundant in BAT, a 4.3 pp difference. Thus, the difference in abundance of H3.2 < K9me2K27me1 > affects a massive 4.3% of the genome when comparing liver and BAT at RT. For reference, the abundance of {K4me3} typically decorates less than 1% of the genome, yet is crucial for active gene transcription [74]. The largest absolute differences with significance are observed with the histone H3.2 proteoforms < K9me3K27me1 > , < K9me2K27me1 > , and < K9me2K27me3K36me1 > . H3.2 < K9me3K27me1 > and < K9me2K27me1 > are more abundant in BAT than in the liver. H3.2 < K9me3K27me1 > is 0.5% abundant in the liver and 3.3% abundant in BAT, affecting 2.7% of the genome. H3.2 < K9me2K27me3K36me1 > is more abundant in the liver than BAT (3.7 and 1.6% of total H3.2, respectively) and affects 2.1% of the genome. In total, these proteoforms are responsible for a shift in 10.8% of the genome between BAT and liver.

Overall, histone H4 PTM changes are observed most frequently at SC (Additional file 4). This includes {K20me1} containing binary combinations and the otherwise unmodified proteoform < K20me1 >, which are more abundant in the liver compared to BAT. Although histone H4 at RT shows no significant differences between tissues for discrete PTMs or binary PTM combinations, proteoform abundances trend toward differences in BAT and liver (Fig. 5B, E). H4 < K20me1 > and < K20me2 > are more abundant in liver than BAT. H4 < K20me1 > is 5.4% abundant in the liver and 4.7% abundant in BAT, changing 0.62% of the genome. H4 < K20me2 > is 39.4% abundant in the liver and 36% abundant in BAT, causing a shift in 3.4% of the genome while only 0.91-fold change. H4 < K20me3 > is more abundant in BAT than liver. H4 < K20me3 > is 19% abundant in the liver and 22% abundant in BAT, causing a shift in 2.5% of the genome. Overall, our results reveal tissue-specific discrete PTM abundances, PTM combinations, and proteoforms for histone H3.2, and, to a lesser extent, H4 at RT housing.

### BAT histone H3.2 changes with cold exposure are centered around K9me2 and K9un

**Fig. 6:**
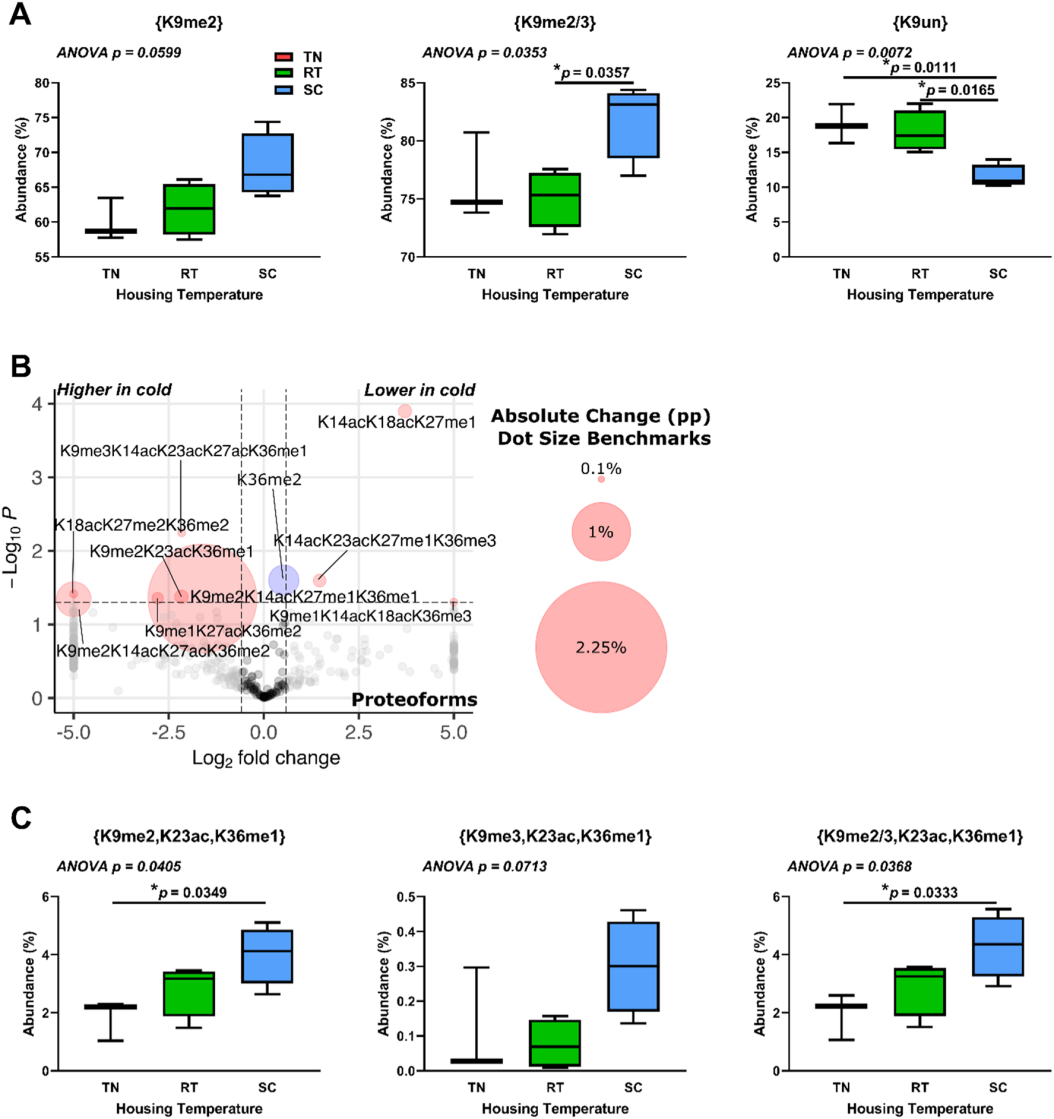
Histone H3.2 K9me2- or K9me3-containing proteoforms increase in brown adipose tissue in response to cold housing. (**A**) Selected discrete H3.2 PTM abundance at different housing temperatures. (**B**) Proteoform changes between SC and TN. Dot size corresponds to absolute percentage point change. Welch’s t-test was used for volcano plot data, with cutoffs of |1.5| for fold change and p < 0.05. Red dots represent both p < 0.05 and fold change >|1.5|; blue dots represent p < 0.05 and fold change <|1.5|; grey dots represent p > 0.05 and fold change >|1.5|; black dots represent p > 0.05 and fold change <|1.5|. (**C**) Ternary (3-PTM) combinations show significant differences with K9 di- and tri-methylation, K23 acetylation, and K36 monomethylation. n = 3–5 per group. One-way ANOVA testing was conducted for all data comparing TN, RT, and SC housing conditions, and p-values are indicated in each plot. pp: percentage point. TN: thermoneutral. RT: room temperature. SC: severe cold

Few temperature-dependent changes in histone H3.2 PTMs and proteoforms were observed in liver (Additional file 5). Our motivating central hypothesis is that there is a BAT-specific epigenetic response to changes in housing temperature. This is rationalized by the importance of histone PTMs in the regulation of transcription and the known unique physiological role of BAT in nonshivering thermogenesis. To determine epigenetic changes that occur during the cold response, we analyzed discrete PTMs, PTM combinations, and proteoforms of histones in BAT from TN-, RT-, and SC-housed mice. Discrete PTM analysis of histone H3.2 shows significant increases in PTMs K9me2 and combined K9me2 and K9me3, {K9me2/3}, and decreased unmodified K9, {K9un}, in response to SC housing (Fig. 6A). Histone H3.2 proteoforms show trends in K9 di-methylation with K36 methylation (Fig. 6B). The presence of K9me2 or K9me3 and K36me1 on the same molecule is greatest at SC when combined with K23ac (Fig. 6C). Supplemental data is available for further analysis (MassIVE: MSV000092105, Additional file 5). Our data indicate a role for altered histone H3.2 methylation and acetylation in the regulation of transcription in BAT in response to cold stress.

### Histone H4 proteoforms in cold-adapted brown adipose tissue show highly specific changes including K16ac

**Fig. 7:**
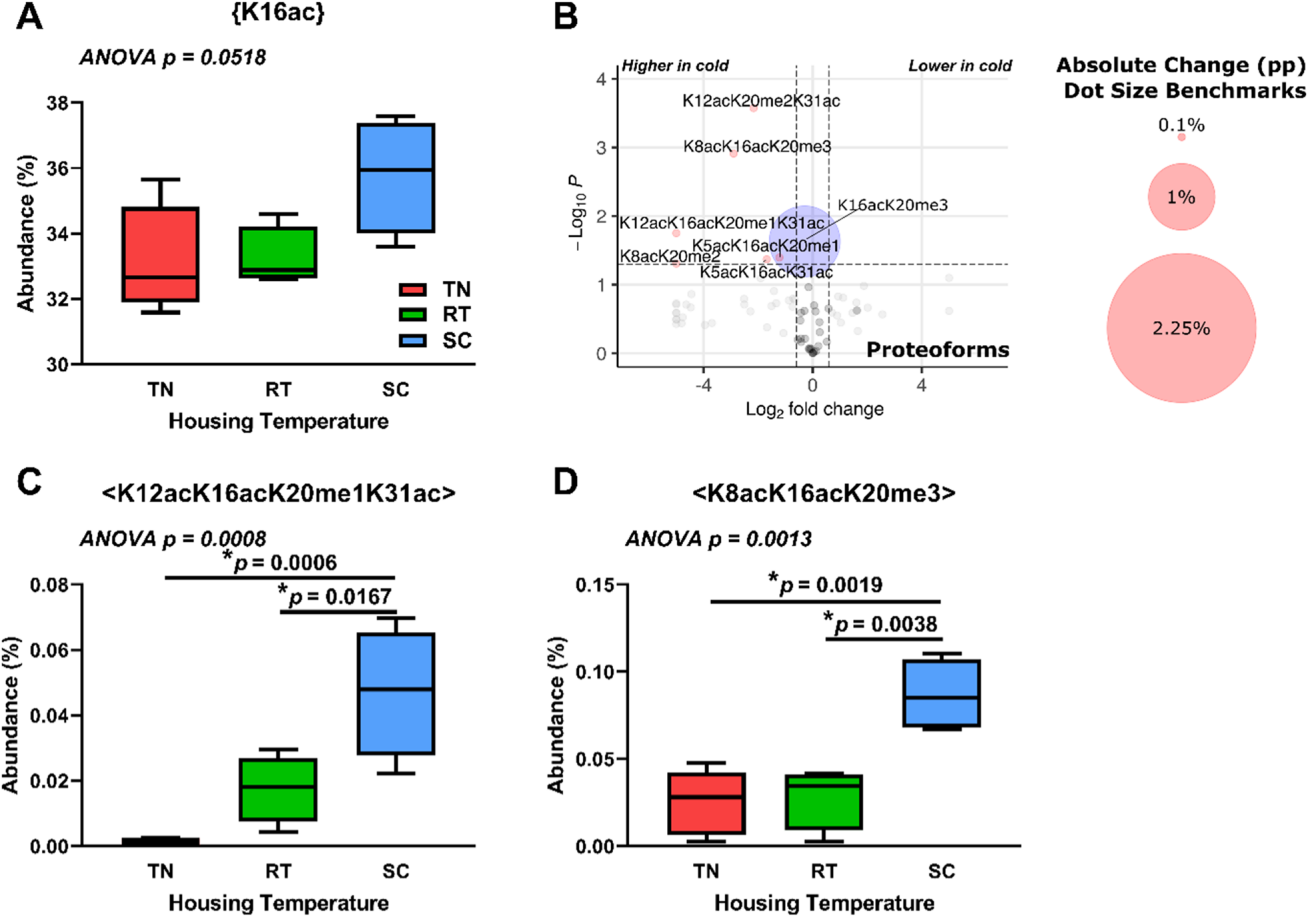
Housing temperature significantly alters histone H4 K16ac-containing proteoforms in brown adipose tissue. (**A**) Discrete K16ac PTM marks on histone H4 at different housing temperatures. (**B**) Histone H4 proteoforms from BAT from SC vs TN housing temperatures. Dot size corresponds to absolute percentage point change. Welch’s t-test was used for volcano plot data, with cutoffs of |1.5| for fold change and p < 0.05. Red dots represent both p < 0.05 and fold change >|1.5|; blue dots represent p < 0.05 and fold change <|1.5|; grey dots represent p > 0.05 and fold change >|1.5|; black dots represent p > 0.05 and fold change <|1.5|. (**C**) H4 < K12acK16acK20me1K31ac > proteoform abundance at different housing temperatures. (**D**) H4 < K8acK16acK20me3 > proteoform abundance at different housing temperatures. n = 4–5 per group. One-way ANOVA testing was conducted for all data comparing TN, RT, and SC housing conditions, and p-values are indicated in each plot. pp: percentage point. TN: thermoneutral. RT: room temperature. SC: severe cold

We hypothesize that there are BAT-specific epigenetic changes with cold exposure that will affect histone H4 proteoforms. As with histone H3.2, we analyzed discrete PTMs, PTM combinations, and proteoforms for histone H4. We observe no significant histone H4 discrete PTM changes between BAT from mice housed at various temperatures. Interestingly, most H4 proteoforms that significantly change with housing temperature include K16ac, which may play a permissive role in the regulation.

of gene expression (Fig. 7A, B) [75, 76]. Further examination reveals specific proteoform changes, which includes < K12acK16acK20me1K31ac > and < K8acK16acK20me3 > , both of which significantly increase at SC vs RT or SC vs TN (Fig. 7C, D). Supplemental data is available for further analysis (MassIVE: MSV000092105, Additional file 6). These results show that specific histone H4 proteoforms increase in BAT in response to chronic cold.

## Discussion

We establish here the first evidence of an epigenetic response to cold adaptation as well as the first measure of the stoichiometric abundance of histone PTMs and proteoforms in BAT. A novel method for quantitative top down proteomics from BAT reveals specific combinations of histone PTMs changing in cis, on the same molecule. A multi-omics approach is used to elucidate and validate these changes. Our proteomic results are supported by analyses of gene expression that indicate changes in epigenetic modifiers, as well as DNA methylation changes, revealing broad epigenetic changes that occur as a result of chronic thermogenic activation. We observe significant changes in the expression of genes that regulate acetyl-CoA levels, one-carbon metabolism, DNA methylation, histone modification, and alternative splicing in response to chronic cold challenge. HCT intersection analysis more specifically identifies the "footprints" of candidate epigenetic writers regulated in response to chronic thermogenic activation. Additionally, our validation of the cold challenge-induced regulatory networks against canonical phenotypic knowledge in the field of thermoregulation enhances their value as a resource for building hypotheses around nodes whose role in thermoregulation is either underappreciated or entirely uncharacterized. Corroborating changes in splicing factor expression, we further uncover and present a number of alternative splice variants that are differentially expressed in response to thermogenic activation. We note decreased promoter and intragenic DNA methylation with colder housing temperatures, and this is associated with reduced *Dnmt1*, *Dnmt3a*, and *Dnmt3b* expression. Consistent with a functional effect of increased expression of acetyl-CoA generating enzymes in BAT, we observe tissue-specific increases in histone H3.2 and H4 total acetylation in response to severe chronic cold. Further examination reveals tissue-specific changes in histone H3.2 and H4 PTMs and proteoforms in BAT in response to chronic thermogenic activation. Our data show for the first time that chronic thermogenic activation results in large-scale epigenetic changes to both DNA and histones in murine BAT that represent both transcriptional “on” and “off” signals. These signals indicate an important role in epigenetic regulation of BAT adaptation to chronic thermogenic activation.

### Housing temperature alters DNA methylation in BAT

We report the first analysis of global thermogenesis-associated DNA methylation changes in BAT. Using RRBS, we discovered that DNA methylation in BAT decreases as thermogenic activation increases. This may result from reductions in DNA methyltransferase activity, as RNA-Seq reveals lower expression of *Dnmt1*, *Dnmt3a*, and *Dnmt3b* at both SC and RT compared to TN [65, 66, 77–79]. The maintenance DNA methyltransferase DNMT1 represses myogenic remodeling of BAT, and inhibits the expression of adiponectin (*Adipoq*) by adipocytes [77, 80]. Additionally, global inhibition of DNMT1 in diabetic obese mice (db/db) in vivo improves whole-body glucose homeostasis in an adiponectin-dependent manner. However, expression of adiponectin, which has been shown to inhibit BAT thermogenesis, is significantly reduced by SC compared with RT or TN housing conditions [43, 81]. We also found no association between *Adipoq* expression and DNA methylation at its promoter, suggesting that DNMT1 activity does not play a major role in the regulation of *Adipoq* expression in BAT during chronic severe cold stress. BAT-specific *Dnmt1*-deletion has no effect on body weight or glucose homeostasis when mice are fed a standard low fat diet [82]. However, thermogenic response to severe cold was not tested in these mice. Both DNMT3a and DNMT3b regulate de novo methylation of DNA and play important roles in adipocyte development and differentiation. DNMT3a regulates preadipocyte differentiation [78]. DNMT3b deficiency in Myf5^+^ brown adipocyte-skeletal muscle precursor cells reduces thermogenic gene expression and upregulates myogenic gene expression in BAT of female mice [79]. Further studies are warranted with regard to the roles of DNA methyltransferases in adult BAT in response to chronic cold.

We also report novel findings from integration of our RRBS data with RNA-Seq data. The genes with the greatest reductions in promoter methylation for which expression is also increased in response to chronic cold stress include *Vldlr*, recently reported to play an important role in fatty acid uptake by thermogenically active BAT, and *Ndufb11*, a component of mitochondrial complex I [83, 84]. Of note, BAT *Vldlr* expression and uptake of VLDL increase in response to cold stress, and VLDLR deficiency impairs thermogenesis in mice [83]. As VLDLR also facilitates the uptake of fatty acids from chylomicrons via lipoprotein lipase-mediated triglyceride hydrolysis, increased *Vldlr* expression represents a means through which cold stress may promote the uptake of fatty acids from circulating chylomicrons postprandially, as well [85]. Our results suggest that altered DNA methylation at promoters is one mechanism through which gene expression for optimal nutrient utilization during thermogenesis is regulated during BAT adaptation to prolonged cold stress.

Methylation of intragenic regions of DNA has been shown to differentially affect the expression of alternative splice variants and modification of histones [68, 86]. Our RNA-Seq analysis yields a number of alternative splice variants that are differentially expressed in BAT at SC vs TN. Additionally, we note associated increased expression of HuR/*Elavl1* and *Igf2bp2*, and decreased expression *Nova2*, *Pspc1*, and many serine-arginine rich splicing factors in BAT from mice housed at SC vs TN. This suggests that expression of RNA-binding proteins known to mediate alternative splicing in adipose tissues is also altered during chronic cold stress [87–89].

Here, using our newly developed method of top-down mass spectrometry-based analysis of histone PTMs and proteoforms in BAT, we also describe a number of histone modifications, particularly increased bulk acetylation, H3.2{K9me2}, and H3.2{K9me3}. DNA methylation has been shown to facilitate the recruitment and methylation of H3K9, and loss of DNMT1 activity is associated with increased acetylation and decreased H3K9 di- and trimethylation, [90–92]. Additionally, H3K9me3 reinforces DNMT1 activity and genome targeting [93]. This suggests that decreased *Dnmt1* expression may play a role in the increased histone acetylation we observe, and perhaps increased H3.2{K9me2} or {K9me3} ensures DNMT1 fidelity with decreased expression. Overall, our results show that chronic cold stress affects DNA methylation in BAT, and that this may play a role in adaptation through effects on transcription, transcript splicing, and histone modifications. One limitation of our study is that RRBS can only assess methylation at 10–15% of all CpG sites in the genome and cannot distinguish between 5-methylcytosine (5mC) and its demethylation intermediate, 5-hydroxymethylcytosine (5hmC) [94–96].

### Our data reveal the first top-down mass spectrometry-based quantitative look at histone modifications in murine BAT

The work described here is the first basal quantitative description and unbiased quantitative statistical analysis of changes in BAT histone PTMs and proteoforms. Top- or middle-down mass spectrometry-based methods are ideal for the unbiased identification and quantitation of histone PTMs [72, 97–102]. PTMs on histones are well-known to affect the binding of downstream factors that effectuate genome function [38, 103, 104]. As described in our recent review, histone modifications often function in concert on single molecules [72]. We have recently published methods to overcome issues in most types of tissue [32, 33]. Here we report the first method for BAT nuclei isolation for subsequent acid extraction of histones and analysis of histone proteoforms by mass spectrometry [34]. A proteoform biology approach brings valuable new insights, such as how multiple PTMs function in concert on single molecules to affect BAT function, while also providing quantitation of more conventional discrete PTMs.

### BAT and liver are epigenetically distinct

Our proteomic analysis of histones H3.2 and H4 in BAT and liver from mice housed at different temperatures reveals tissue-specific responses to cold. Histones in BAT are more acetylated at SC compared to other housing temperatures. In liver, there is no significant effect of housing temperature on histone acetylation levels. Additionally, there is no difference in bulk histone acetylation levels between tissues. Although our focus for comparison of proteoforms in these tissues was RT housing temperature, we observe the greatest number of significant differences in single PTMs, 2-PTM combinations, and proteoforms between tissues at SC. The significantly differing combinations containing H3.2 are always more abundant in BAT compared to liver, indicating transcriptional repression of genes through HP1 in BAT [105]. The abundance of H3.2{K36un} and combinations including K36un are typically more abundant in BAT compared to liver, consistent with decreased deposition of K36 methylation during transcription [106]. Thus, there is more K9 methylation to recruit HP1, which would result in less transcription and less co-transcriptional methylation of K36. Fewer differences between tissues are observed with histone H4 but center on {K20me1} at SC. H4{K20me1} binary combinations and H4 < K20me1 > are more abundant in the liver. Generally, we observe PTM differences between tissues that indicate less active transcription in BAT than in liver, and these differences are most pronounced under severe chronic cold housing conditions.

At RT, histone H3.2 proteoforms that are significantly more abundant in BAT than liver are H3.2 < K9me2K27me1 > , H3.2 < K9me3K27me1 > , H3.2 < K9me3 > , and H3.2 < K9me2 > and indicate an increase in heterochromatin in BAT [104, 107]. Histone H4 proteoforms H4 < K20me3 > , H4 < K16acK20me2 > , and H4 < K16acK20me3 > trend toward greater abundance in BAT compared to liver. These proteoforms indicate transcriptional repression or a poised state [108]. H4 < K20me2 > and H4 < K20me1 > are significantly more abundant in liver than BAT and are associated with transcriptional activation [109]. H4 < K20me2 > is 39% of all histone H4 in liver and 36% abundant in BAT, affecting 3.4% of the genome. H4 < K20me1 > is 5.4% of all histone H4 in liver and 4.7% abundant in BAT. Significant discrete changes between tissues are observed in histone H3.2 but not histone H4 at RT. The changes in proteoform abundances reflect poised positions that act as a scaffold for other factors that determine downstream effects [38]. These proteoforms are often quite abundant in tissues and cultured cells and their shift indicates a broad difference between tissue epigenetic regulation [34, 38]. Significantly changing proteoforms of histone H3.2 and H4 indicate higher levels of heterochromatin in BAT compared to liver at RT [104, 107–109]. Our histone proteoform findings also suggest that fewer genes are being actively transcribed in BAT under this condition of mild chronic cold activation.

It is important to keep in mind differences in tissue heterogeneity when making comparisons between tissues or generalizing findings to a specific tissue. Prior work has established that, even under pro-inflammatory, obesogenic diet feeding conditions, BAT consists primarily of brown adipocytes, with immune cells comprising less than 5% of all live cells when mice are housed at RT [110]. Chronic cold exposure increases the proliferation of brown adipocytes and immune and vascular endothelial cells [111, 112]. In liver, approximately one-third of cells are non-parenchymal and there is tissue zonation [113, 114]. Differences in cellular transcriptional profiles and DNA methylation have already been demonstrated in the context of hepatocyte zonation [115]. Thus, in each tissue, particular cells and cell-types may exhibit differential epigenetic landscapes that change depending on location in the tissue and environmental stimuli.

### Housing temperature affects histone modifications in BAT

Our data show that BAT responds epigenetically to cold stimuli and further reveal changes in its epigenome that may drive its function during cold adaptation. Bulk histone acetylation increases in BAT from SC- vs TN-housed mice. This is consistent with changes in gene expression upon cold challenge. Expression of acetyl-coenzyme A (CoA) synthetase short chain family, member 2 *(Acss2),* carbohydrate response element binding protein * (Chrebp/Mlxipl),* and ChREBP target adenosine triphosphate (ATP) citrate lyase *(Acly)* increase in BAT when mice are housed under chronic mild and severe cold conditions [43, 116]. ACLY and ACSS2 provide acetyl-CoA for histone acetylation through citrate and acetate, respectively, and their upregulation provides potential mechanisms for our findings. Additionally, carnitine acetyltransferase (CrAT) and carnitine octanoyltransferase (CrOT) have both been shown to play important roles in the ACLY- and ACSS2-independent conversion of short- and medium-chain acetylcarnitines to acetyl-CoA [117, 118]. Analysis of RNA-Seq data reveals that both *Crat* and *Crot* expression increase in BAT during chronic cold housing, suggesting additional mechanisms through which BAT activation generates acetyl-CoA that can be used for post-translational modification of histones. We also observe a significant cold-induced transcriptional footprint of histone acetyltransferases CBP/p300 in BAT. CBP/p300 are generally considered to be the most active histone acetyltransferases and play a crucial role in regulating adipocyte plasticity [52]. CBP and p300 both preferentially acetylate histones H3 and H4 [32, 119]. Indeed, histone H4 and H3.2 acetylation sites are the most occupied at SC in BAT, indicating a shift in BAT acetyl-CoA pools. Taken together, our findings are consistent with increased intracellular acetyl-CoA levels and histone acetylation via p300/CBP under conditions of chronic cold stress.

Specific histone post-translational modifications (PTMs) additionally suggest a mechanism through which temperature-dependent selective transcription in BAT may occur. Further investigation into the K9me2 mark reveal interplay with K36 methylation (K36me1 and K36me2) and such combinations enable K9me2 to affect transcriptional repression of some genes while others are transcribed [106]. The decrease in {K9un} supports this model. While the lack of commercially available {K9un} antibodies means there are few studies implicating function, a reasonable interpretation of loss of {K9un} is a shift away from a more transcriptionally permissive but still poised state. {K9un} is directly prone to either acetylation (activation) or methylation (repression) without erasure of the antagonistic mark [107, 120]. As thermogenic activation increases, histone H4 proteoforms with K16ac increase. Importantly, {K16ac} acts as a scaffold and recruits factors for transcriptional activation [72]. Mechanistically, H4{K20me2} promotes {K16ac} and further rapid acetylation in *cis* at K12, K8, and K5 [38]. In support of these PTM data, we note increased expression and transcriptional footprints of *Kat8* with thermogenic activation between SC- and RT- vs TN housing temperatures. KAT8 forms a specific H4K16 acetyltransferase complex with MSL1, MSL2, and MSL3 [121–123]. While a role for KAT8 in the thermogenic function of brown adipocytes has not been tested, KAT8 is required for adipocyte differentiation prior to clonal expansion in an in vitro culture model, [124]. Taken together, histone H3 PTMs indicate persistent transcriptional repression of certain genes (through methylation) and histone H4 PTMs suggest an overall transient or poised state of transcription (through acetylation) in BAT when mice are housed at colder temperatures. These findings suggest a mechanism in BAT through which thermogenic activation fine-tunes transcription in a nutrient-sensitive manner via increased acetyl-CoA and KAT8 activity to modulate histone methylation-dependent gene repression.

Our gene expression and transcriptional footprint analyses suggests a regulatory mechanism of H3K9 methylation-dependent gene repression in BAT. RNA-Seq analysis reveals that *Fcor* expression increases with decreasing housing temperature. FCOR regulates FOXO1 activity through repressive acetylation [125]. We also observe an increased SIRT6 transcriptional footprint under colder housing temperatures. SIRT6 deacetylates FOXO1, and SIRT1 deacetylates SIRT6, thus increasing SIRT6 activity [126, 127]. When not acetylated, FOXO1 forms a complex with SIRT1 and mediates the expression of SIRT1 [125, 128]. Both SIRT1 and SIRT6 are known H3{K9ac} deacetylases [129]. Deacetylation of H3K9 allows for methylation [120]. Also, SIRT1 mediated deacetylation of SUV39H1 at K266, activates this H3K9 dimethyltransferase, generating H3K9me3 [104]. The KDM6C footprint also increases upon cold challenge, which specifically demethylates H3K9me3 to K9me2 [8]. Thus, dramatically higher levels of {K9me2} at SC likely result from a transcriptionally repressive feedback loop with {K9me2/3} with cold challenge.

## Conclusions

In conclusion, with the addition of data from our newly developed method for top-down mass spectrometry-based proteomic quantitation of histone proteoforms in BAT, we establish the first evidence of tissue-specific epigenetic responses to severe chronic cold stress in that tissue. The transcriptional footprints of many histone modifying proteins, including both writers and erasers, increase upon cold challenge. The reduction in promoter DNA methylation and increased histone acetylation both suggest increased transcription, while increased histone H3.2 K9 di- and trimethylation indicates transcriptional repression under chronic cold conditions. These epigenetic changes, as well as alternative splicing, support an important role in adaptation to cold stress in BAT by regulating transcript expression. Significant work remains to understand the epigenetic changes established here. Future work will elucidate cell type specificity of these changes and the detailed gene-specific mechanisms of epigenetic regulation of BAT adaptation to chronic thermogenic activation.

### Supplementary Information

**Additional file 1:** High confidence transcriptional target (HCT) intersection analysis dataset. WT: wild type. TN: thermoneutral. RT: room temperature. SC: severe cold.

**Additional file 2**: Alternative splice variants in BAT from severe cold vs thermoneutral housed mice. A3SS: alternative 3’ splice site. A5SS: alternative 5’ splice site. MXE: mutually exclusive exons. RI: retained intron. SE: skipped exon.

**Additional file 3**: RNA-Seq differentially expressed gene (DEG) reanalysis and RRBS differentially methylated gene (DMG) and differentially methylated promoter (DMP) analyses. TN: thermoneutral. RT: room temperature. SC: severe cold.

**Additional file 4**: Summary and statistical analysis of differences between BAT and liver histone H3.2 and H4 post-translational modifications (PTMs), binary PTM combinations, and proteoforms in each housing temperature. TN: thermoneutral. RT: room temperature. SC: severe cold.

**Additional file 5**: BAT and liver histone H3.2 discrete post-translational modifications (PTMs), binary PTM combinations, and proteoform percent abundance for each biological replicate. TN: thermoneutral. RT: room temperature. SC: severe cold.

**Additional file 6: **BAT and liver histone H4 discrete post-translational modifications (PTMs), binary PTM combinations, and proteoform percent abundance for each biological replicate. TN: thermoneutral. RT: room temperature. SC: severe cold.

**Additional file 7: Table S1.** Average body weights, fat mass, lean mass, tissue weights, and nonfasting glucose of male C57BL/6J mice after exposure to thermoneutral or chronic cold housing temperatures. Results shown are mean ± SEM. n = 20 per group. One-way ANOVA testing was conducted for each parameter, and p-values are indicated as follows: *p < 0.05 for SC vs TN, #p < 0.05 for SC vs RT and TN, and @p < 0.05 for RT vs TN. BAT: brown adipose tissue. gWAT: gonadal white adipose tissue. TN: thermoneutral. RT: room temperature. SC: severe cold.

**Additional file 8: Table S2.** Results of method optimization of histone isolation from BAT. Color scheme indicates the highest yield of histones in green (2) and the lowest yield of histones in red. Mass histone is calculated using offline HPLC peak area and standard curve.

**Additional file 9: Table S3.** Mass of histone H3.2 and H4 obtained from **(a)** BAT and **(b)** liver at different housing temperatures. Mass histone is calculated using offline HPLC peak area and standard curve. Results shown are mean ± SEM. n = 4–5 per group. TN: thermoneutral. RT: room temperature. SC: severe cold.

## Data Availability

The RNA-Seq dataset reanalyzed during this study was obtained from a published article and its supplementary files [43]. The RRBS datasets generated and analyzed during the current study are available in GEO (https://www.ncbi.nlm.nih.gov/geo/query/acc.cgi?acc=GSE234588). The histone H3.2 and H4 mass spectrometry datasets generated and analyzed during the current study are uploaded to the MassIVE repository (ftp://massive.ucsd.edu/MSV000092105/). All other data described in this manuscript are contained within the manuscript or as associated Additional files.

## Abbreviations

ACN: Acetonitrile
AEBSF: 4-Benzenesulfonyl fluoride hydrochloride
AGC: Automatic gain control
ANOVA: Analysis of variance
BAT: Brown adipose tissue
C3: 3 Carbon chain
CBP: CREB-Binding Protein
ChIP-Seq: Chromatin immunoprecipitation sequencing
DEG: Differentially expressed gene
DMP: Differentially methylated promoter
DNA: Deoxyribonucleic acid
DNMT1: DNA methyltransferase 1
DNMT3A: DNA methyltransferase 3A
DNMT3B: DNA methyltransferase 3B
DTT: Dithiothreitol
ETD: Electron transfer dissociation
FA: Formic acid
FC: Fold change
FCOR: FOXO1-corepressor
FDR: False discovery rate
FOXO1: Forkhead Box O1
EHMT2: Euchromatic histone-lysine N-methyltransferase 2
GluC: Endoproteinase Glu-C from Staphylococcus aureus Protease V8
HCT: High confidence transcriptional target
HDAC: Histone deacetylase
HPLC: High-performance liquid chromatography
K: Lysine
Kat8: Lysine Acetyltransferase 8
Kdm2b: Lysine demethylase 2B
Kdm3a: Lysine demethylase 3A
Kdm5b: Lysine demethylase 5B
Kdm5c: Lysine demethylase 5C
Kdm8 (JMJD5): Lysine demethylase 8
LC–MS/MS: Liquid chromatography tandem mass spectrometry
MACS2: Model-based analysis of ChIP-Seq
MS: Mass spectrometry
MS2: Tandem mass spectrometry or mass spectrum
mzXML: File format, eXtensible markup language
NIB: Nuclei isolation buffer
NP-40: Nonyl phenoxypolyethoxylethanol
NST: Nonshivering thermogenesis
OR: Odds ratio
P300: E1A-Binding Protein, 300-KD
PTM: Post-translational modification
RNA-Seq: RNA sequencing
RRBS: Reduced representation bisulfite sequencing
RT: Room temperature
SC: Severe cold
SEM: Standard error of the mean
Setd7: Histone-lysine *N*-methyltransferase SETD7
SIRT1: Sirtuin-1
SMYD3: SET and MYN-domain containing 3
SRA: Sequence read archive
SUV39H1: Histone-lysine *N*-methyltransferase SUV39H1
TN: Thermoneutral
